# Hybrid Symbiotic Organisms Search Optimization Algorithm for Scheduling of Tasks on Cloud Computing Environment

**DOI:** 10.1371/journal.pone.0158229

**Published:** 2016-06-27

**Authors:** Mohammed Abdullahi, Md Asri Ngadi

**Affiliations:** 1 Department of Computer Science, Universiti Teknologi Malaysia, 81310 Johor Bahru, Malaysia; 2 Department of Mathematics, Ahmadu Bello University, Zaria, Nigeria; University of Vermont, UNITED STATES

## Abstract

Cloud computing has attracted significant attention from research community because of rapid migration rate of Information Technology services to its domain. Advances in virtualization technology has made cloud computing very popular as a result of easier deployment of application services. Tasks are submitted to cloud datacenters to be processed on pay as you go fashion. Task scheduling is one the significant research challenges in cloud computing environment. The current formulation of task scheduling problems has been shown to be NP-complete, hence finding the exact solution especially for large problem sizes is intractable. The heterogeneous and dynamic feature of cloud resources makes optimum task scheduling non-trivial. Therefore, efficient task scheduling algorithms are required for optimum resource utilization. Symbiotic Organisms Search (SOS) has been shown to perform competitively with Particle Swarm Optimization (PSO). The aim of this study is to optimize task scheduling in cloud computing environment based on a proposed Simulated Annealing (SA) based SOS (SASOS) in order to improve the convergence rate and quality of solution of SOS. The SOS algorithm has a strong global exploration capability and uses fewer parameters. The systematic reasoning ability of SA is employed to find better solutions on local solution regions, hence, adding exploration ability to SOS. Also, a fitness function is proposed which takes into account the utilization level of virtual machines (VMs) which reduced makespan and degree of imbalance among VMs. CloudSim toolkit was used to evaluate the efficiency of the proposed method using both synthetic and standard workload. Results of simulation showed that hybrid SOS performs better than SOS in terms of convergence speed, response time, degree of imbalance, and makespan.

## Introduction

Cloud computing is one of the recent developments in the field of computing which enables limitless usage of Information Technology in diverse domains such as medicine, business, mobile system, smart systems, environmental computing etc [1, 2]. This has lead to rapid adoption of cloud computing in recent years, because it acts as an efficient computing paradigm for renting Information Technology (IT) services and infrastructures based on pay-per-use model [3]. Pay-per-use model eliminates the need for companies to invest in acquisition of IT infrastructures or software licenses.

Cloud services are categorized as Software as a Service (SaaS), Platform as a Service (PaaS), and Infrastructure as a Service (IaaS) [4]. These services are provisioned to users of virtual resources which make cloud computing resources dynamic and elastic thereby creating the notion of unlimited resources. User are charged for the services they consumed on pay-per-use basis, and this flexible mode of charging users has encouraged migration of IT services to the cloud environment. The focus of this study in on IaaS cloud where computing resource are offered as services. Users subscribed for VMs for execution of their tasks, and better utilization of physical resources is directly dependent on the optimal scheduling of tasks on VMs.

Task scheduling has been one of the widely researched problems in cloud computing, but it remains a NP-hard problem [5]. Pool of Virtual resources are made available to cloud users by network of servers in IaaS layer. IaaS layer delivers hardware and associated software which enable provision of flexible and efficient computational capacities to end users. The resource management subsystem of IaaS layer is responsible for scheduling submitted tasks for execution. Scheduling of tasks on VMs is a key process on IaaS cloud, because mapping of tasks to VMs need to be carried out in an efficient manner due to heterogeneous and dynamic characteristics of VMs. Since there is no exact algorithm for finding optimal solution for NP-Complete problems, a good schedule solution can only be achieved via heuristic methods [6–9]. The objective of task scheduling algorithm is to reduce execution time and cost; the algorithm decides which VM should execute the received task. In cloud computing environment, VMs have heterogeneous processing capacities and characteristics. Therefore, load balancing among VMs needs to be taken into account when scheduling tasks, which entails careful coordination and optimization in order achieve lower makespan [10, 11]. Task scheduling algorithms try to efficiently balance the load of the system taking into consideration total execution time of available VMs.

Methods proposed in the literature for solving task scheduling problems are either heuristic based or metaheuristic based. Heuristic based methods try to find optimal solution based on some predefined rules, and the quality of solutions obtained by these methods are dependent on the underlining rules and problem size. The solution obtained by heuristics search methods are not feasible and they are generated at high operating cost [12].

Metaheuristic techniques have been extensively applied to solve optimization problems. Metaheuristic methods employ a pool of candidate solutions to traverse solution space unlike the mathematical and heuristic techniques that uses single candidate solution. This attribute of metaheuristic algorithms make them perform better than mathematical and heuristic techniques. Some of the popular metaheuristic methods for solving task scheduling problems in cloud computing environment are Genetic Algorithm (GA) [13], Particle Swarm Optimization (PSO) [14], Ant Colony Optimization (ACO) [15, 16], League Championship Algorithm (LCA) [11], BAT algorithm [17, 18], Symbiotic Organisms Search (SOS) [10]. The idea of SOS as a metaheuristic algorithm was introduced in [19]. SOS algorithm was inspired by interactive relationship exhibited by organisms in ecosystem for survival and it was shown to perform competitively well [19] with GA, Differential Evolution (DE), PSO, Honey Bee Colony (HBC). Since the introduction of SOS algorithm, a number of researches have applied SOS to solve some practical optimization problems [10, 20–25]. Therefore, the potential of SOS in finding global solution to optimization problems exhibited so far make it attractive for further investigation and exploration.

Quality of solution and convergence speed obtained by metaheuristic algorithms can be improved by its hybridization with either a metaheuristic algorithm or local search method, by generating initial solution using heuristic search techniques or by modifying the transition operator [26]. To the best of our knowledge none of the aforementioned techniques have been explored to investigate the possible improvement of SOS in terms of convergence speed and quality of solution obtained by SOS.

In this paper, we developed a fitness function model for computing makespan taking into account utilization of VMs in order to reduce degree of imbalance among VMs. We studied task scheduling using Improved Symbiotic Organism Search(SASOS). The proposed SASOS combines SA method [27] and SOS optimization algorithm [10]. The SOS uses fewer control parameters, and has a strong exploration and faster convergence capability. SA was used to search local solution space identified by SOS which equip SASOS with exploitative ability. The objective is to obtain optimal schedules by minimizing makespan and degree of imbalance among VMs.

The main contributions of the paper are:

1. An objective function for optimum scheduling of tasks on VMs is presented taking into account the utilization level of VMs in order to reduce makespan, response time and degree of imbalance among VMs.
2. Hybridization of SOS with SA, applying SA to find optimum solution in the global solution regions identified by SOS.
3. Implementation of the proposed method in CloudSim.
4. Performance comparison of SOS and the proposed method in terms of makespan, response time and degree of imbalance.
5. Empirical analysis of convergence speed obtained by SASOS and SOS.

The organization of remaining parts of the paper is as follows. Metaheuristic algorithms applied to task scheduling problems in cloud and SOS are presented in Section 2. Section 3 describes problem formulation. Design of proposed algorithm and its description is presented in Section 4. Results of simulation and its discussion are in Section 5. Section 6 presented summary and conclusion of the paper.

## Related Works

Metaheuristic algorithms [10, 11, 13–18] have been applied to solve task assignment problems in order to reduce makespan and response time. These methods have proven to find optimum mapping of workloads to resources which reduces cost of computation, better quality of service, and increased utilization of computing resources. ACO, PSO, GA, and their variants are the mostly used nature inspired population based algorithms in the cloud. PSO outperforms GA and ACO in most situations [28] and has faster execution time. PSO is simpler to implement as compared to GA and ACO respectively. Workflow scheduling problems have been widely studied using PSO [14, 29–31] with aim of reducing communication cost and makespan. Scheduling of Independent tasks have also been studied in cloud using PSO [32–34] and it proved to ensure minimal makespan. Improved and hybrid versions [32, 35, 36] of PSO were also proposed for scheduling of tasks in cloud and they obtained better solution than those of ACO and GA. Recently, discrete version of SOS was applied to task scheduling problem in cloud computing environment [10] and SOS algorithm was found to outperform PSO and its popular variants.

### Symbiotic Organisms Search

The SOS algorithm was inspired by symbiotic interactions between paired organisms in an ecosystem. Each organism denotes a potential solution to an optimization problem under consideration and has its position in the solution space. Organisms adjust their position according to mutualism, commensalism, and parasitism biological interaction models of the ecosystem. With mutualism form of interaction, the two interacting organisms benefit from the relationship and this is applied in the first phase of the algorithm. The commensalism association enables only one organism to benefit from the relationship while other is not harmed. The commensalism association is applied in the second phase of the algorithm to fine tune the solution space. With parasitism interaction, only one organism benefits while the other is harmed. The parasitism interaction technique is applied in the third phase of the algorithm. The fittest organisms survive in the solution space while the unfit ones are eliminated. The best organisms are identified as those that benefited from the three phases of the interaction. The phases of the procedure are continuously applied on the population of organisms which represents candidate solutions until the stopping criterion are reached. The basic pseudocode of SOS is presented in Algorithm 1. The quality of position of the organism depends on the fitness which defines the extent of adaptation of the organisms to the ecosystem.

SOS share some common features with most of the nature inspired algorithms. The candidate solutions are represented by population of organisms using operators to direct the search process by candidate solutions. Selection mechanism is used to keep better solutions, and it requires the settings of population size and stopping criterion before the search process starts. On the other hand, SOS does not require algorithm specific parameters unlike PSO that needs inertia weight, social and cognitive factors or GA that used crossover and mutation. Inadequate turning of these algorithm specific parameters could lead to non-optimal solutions.

**Algorithm 1** *The basic pseudo code for SOS*

**INPUT**: Ecosize, Initial population, Stopping criteria

**OUTPUT**: Optimal solution

1:  **Do**

2:   **For** (All organisms in the ecosystem)

3:    Determine the best organism

4:    Mutualism Phase

5:    Commensalism Phase

6:    Parasitism Phase

7:   **End For**

8:  **While** stopping condition is not exceeded

The SOS algorithm procedure starts with a randomly generated population of organisms called ecosystem. Then, the positions of the organisms are updated using the three phases of SOS. In D-dimensional solution search space, a search population of *n* organisms is denoted as *X* = {*X*_1_, *X*_2_, *X*_3_, …, *X*_*n*_}. The position of the *ith* organism is denoted as *X*_*i*_ = {*x*_*i*1_, *x*_*i*2_, *x*_*i*3_, …, *x*_*id*_}. A fitness function is defined to determine the quality of solution obtained by an organism. Each organism represents a task schedule which is encoded in a vector of 1x*n* dimension, where *n* is the number of tasks. The elements of the vector are natural numbers in the range [0, *m* − 1], where *m* is the number of resource for executing the tasks. The best position searched by all organisms so far is represented by *X*_*best*_. Since task scheduling is a discrete optimization problem and SOS was originally proposed to solve a continuous optimization problem, we adapt a function proposed in [10] for mapping continuous position to a discrete position. The mapping function is defined in Eq (1). The fitness value of each organism is evaluated iteratively using their corresponding positions as input in the three phases of the algorithm as explained in the following subsections.

$$\begin{array}{c} X_{i}=round\left(X_{i}\;mod\;m\right) \end{array}$$ (1)

where *m* is the number of resources for executing the submitted tasks.

#### Mutualism Phase

Suppose *X*_*i*_ is the *ith* member of the ecosystem. In this phase, *X*_*j*_ is randomly selected from the swarm of organisms to interact with *X*_*i*_, *i* ≠ *j* for mutual benefit. The essence of the interaction is to improve extent of survival of both *X*_*i*_ and *X*_*j*_ in the ecosystem. The new candidate solutions for *X*_*i*_ and *X*_*j*_ are obtained according to Eqs (2) and (3).

$$\begin{array}{c} X_{i}^{*}=X_{i}+U\left(0,1\right)*\left(X_{best}+MV*\alpha \right) \end{array}$$ (2)

$$\begin{array}{c} X_{j}^{*}=X_{j}+U\left(0,1\right)*\left(X_{best}+MV*\beta \right) \end{array}$$ (3)

$$\begin{array}{c} MV=\frac{1}{2}\left(X_{i}+X_{j}\right) \end{array}$$ (4)

where *i* = 1, 2, 3, …, *ecosize*; *j* ∈ {1, 2, 3, …, *ecosize*|*j* ≠ *i*}; *U*(0, 1) is a vector of uniformly distributed random numbers between 0 and 1. *MV* is the mutual relationship vector between *X*_*i*_ and *X*_*j*_ as defined in Eq (4). *X*_*best*_ represents the organism with best fitness value. *α* and *β* represent the benefit factors between organism *X*_*i*_ and *X*_*j*_. In a mutual relationship, an organism might benefit heavily or lightly while interacting with a mutual partner. Therefore, *α* and *β* are stochastically obtained as either 1 or 2. 1 and 2 denotes light and heavy benefits respectively. The new candidate solutions replaced the old ones if their fitness values are better than those of the old ones. In this case, $X_{i}^{*}$ and $X_{j}^{*}$ replace *X*_*i*_ and *X*_*j*_ respectively in the next generation of ecosystem. Otherwise, $X_{i}^{*}$ and $X_{j}^{*}$ are discarded while *X*_*i*_ and *X*_*j*_ survives to the next generation of the ecosystem. This scenario is captured by Eqs (5) and (6).

$$\begin{array}{c} X_{i}=\{\begin{array}{cc} X_{i}^{*} & \;\text{if}\;f\left(X_{i}^{*}\right)>f\left(X_{i}\right)\\ X_{i} & \;\text{if}\;f\left(X_{i}^{*}\right)\leq f\left(X_{i}\right) \end{array} \end{array}$$ (5)

$$\begin{array}{c} X_{j}=\{\begin{array}{cc} X_{j}^{*} & \;\text{if}\;f\left(X_{j}^{*}\right)>f\left(X_{j}\right)\\ X_{j} & \;\text{if}\;f\left(X_{j}^{*}\right)\leq f\left(X_{j}\right) \end{array} \end{array}$$ (6)

where *f*(.) denotes the fitness evaluation function.

#### Commensalism Phase

In commensalism phase, an *ith* member of the ecosystem randomly selects an organism *X*_*j*_ for interaction with *X*_*i*_, *i* ≠ *j*. In this case, *X*_*i*_ intends to benefit from *X*_*j*_, and *X*_*j*_ neither gaining or losing from the interaction. The interaction is mathematically modelled by Eq (7).

$$\begin{array}{c} X_{i}^{*}=X_{i}+U\left(-1,1\right)*\left(X_{best}+X_{j}\right) \end{array}$$ (7)

where *U*(−1, 1) is a vector of uniformly distributed random numbers between −1 and 1. *X*_*best*_ represents the organism with best fitness value similar to that of mutualism phase. *X*_*i*_ is updated to $X_{i}^{*}$ as computed in Eq (7), if the fitness value $f(X_{i}^{*})$ is better that of *f*(*X*_*i*_). The relationship for updating *X*_*i*_ is given by Eq (8).

$$\begin{array}{c} X_{i}=\{\begin{array}{cc} X_{i}^{*} & \;\text{if}\;f\left(X_{i}^{*}\right)>f\left(X_{i}\right)\\ X_{i} & \;\text{if}\;f\left(X_{i}^{*}\right)\leq f\left(X_{i}\right) \end{array} \end{array}$$ (8)

#### Parasitism Phase

In parasitism phase, an artificial parasite called parasite vector is created by cloning an *ith* organism *X*_*i*_ and modifying it using randomly generated number. Then, *X*_*j*_ is randomly selected from ecosystem, and fitness values of parasite vector and *X*_*j*_ are computed. If the parasite vector is fitter than *X*_*j*_, *X*_*j*_ is replaced by the parasite vector, otherwise *X*_*j*_ survives to the next generation of ecosystem and parasite vector is discarded. *X*_*j*_ is updated according to the relation in Eq (9).

$$\begin{array}{c} X_{j}=\{\begin{array}{cc} PV & \;\text{if}\;f\left(PV\right)>f\left(X_{j}\right)\\ X_{j} & \;\text{if}\;f\left(PV\right)\leq f\left(X_{j}\right) \end{array} \end{array}$$ (9)

*PV* denotes the parasite vector.

### Simulated Annealing

Simulated annealing was introduced in [37, 38]. It is a simple and robust optimization method inspired by the annealing process of physical systems. When a solid is heated, there will be disorderliness in the state of particles as the temperature and internal energy increases. As the cooling process is applied, the particles are ordered. The internal energy is minimized as the temperature reached the room state. The simulated annealing algorithm is obtained by using the internal energy as the objective function and temperature evolution as the control parameter [39]. The SA procedure begin with an initial solution *X* and created an updated solution *X*′ in the neighborhood of the current solution *X*. The algorithm will generate a solution if the fitness value *F*(X*) is lower than *F*(*X*). However, a higher fitness of X* is accepted at times with the probability define in Eq (10). This strategy enables the search procedure to avoid entrapment in local optima.

$$\begin{array}{c} P_{r}=\exp\left(\frac{-(f\left(X^{*}\right)-f\left(X\right))}{T}\right) \end{array}$$ (10)

where *F*(X*) and *F*(*X*) are the fitness evaluation functions of neighbor and current solutions respectively; and *T* is the temperature known as the control parameter. The algorithm perform such series of moves in order to attain equilibrium state. The temperature parameter is determined according to cooling rate. The cooling rate used in this work is adopted from [28] as defined in Eq (11). The algorithm terminates if there is no further improvement after series of decrease in temperature. The temperature mainly affects the global search performance of SA algorithm. At high initial value of temperature, the SA will have a high chance of locating global optimal solution which in consequence increase the computation time. Conversely, when the value of initial temperature is low, the chance of algorithm locating global optimal solution is limited though the computation time of the algorithm will be shorter.

$$\begin{array}{c} T=\delta^{i}*T_{o}+T_{f} \end{array}$$ (11)

where *δ*^*i*^ is the temperature descending rate, 0 < *δ* < 1, *i* is the number of times neighbor solutions have been generated so far; *T*_*o*_ is the initial temperature; *T*_*f*_ is the final temperature. The basic structure of SA algorithm is presented as Algorithm 2.

**Algorithm 2** *The basic pseudo code for SA*

**INPUT**: Initial Temperature, Final Temperature, Cooling rate

**OUTPUT**: Best solution

1:  Generate an initial solution *x*_0_

2:  **Do**

3:   Generate new solution *x*′ in the neighborhood of *x*_0_

4:   Compute the acceptance probability *P*_*r*_ according to Eq (10)

5:   Decide on acceptance or rejection of new solution based on *P*_*r*_.

6:   Memorize the best solution found so far

7:   Reduce the temperature

8:  **While** stopping condition is not exceeded

### Simulated Annealing based Symbiotic Organisms Search (SASOS)

With proposed SASOS, the new candidate solutions are generated by moving the previous solution towards another randomly selected solution from ecosystem using the three phases of SOS as modelled by Eqs (2) (3) and (7). The techniques increase the chance of locating the global optima, but it cannot ensure a better solution, so convergence rate is slow. The convergence will be faster, if every new candidate solution is better than the previous one. However, if only the better solutions are accepted as described in Eqs (5) (6) (8) and (9), the algorithm could be entrapped in local optima. Therefore, to improve the speed of convergence and quality of solution, SA technique is employed into solution search procedure of mutualism and commensalism phases of the SOS. The parasitism phase is left untouched because it perturbates the solution space by deleting the inactive solutions and injecting the active ones which could move the search procedure out of the local optima region. The SA can obtain better solution for the best organism of each generation of organisms, and accept poor neighbor solutions using certain probability. In each iteration, the possibility of accepting poor solutions are higher at the early stage of the search process but reduces at the later stage of the search process. The SA probability of accepting neighbor solution into new generation of organisms is given in Eq (12).

$$\begin{array}{c} P_{r}=exp\left(-\frac{f\left(X_{i}^{*}\right)-f\left(X_{i}\right)}{T}\right) \end{array}$$ (12)

where $f(X_{i}^{*})$ and *f*(*X*_*i*_) are the fitness evaluation functions of neighbor and current solutions respectively, *T* is the control parameter. The pseudocode for SA used in SASOS is described in Algorithm 3. The steps of SASOS are described in Algorithm 4.

**Algorithm 3** *SA Procedure based on SOS solution search*

1:  **Do**

2:   Generate new solution $X_{i}^{*}$ in the neighborhood of *X*_*i*_ based on specified solution search eqaution(i.e Eqs (2) and (3) for mutualism phase; Eq (7) for commensalism phase)

3:   $\Delta f=f\left(X_{i}^{*}\right)-f\left(X_{i}\right)$

4:   **If** Δ*f* ≤ 0 **or** $\exp\left(-\frac{\Delta f}{T}\right)>U\left(0,1\right)$

5:    $X_{i}\leftarrow X_{i}^{*}$

6:   **End If**

7:   *U*(0, 1) is a uniformly random generated number between 0 and 1

8:  **While** stopping condition is not exceeded

**Algorithm 4** *The pseudo code for SASOS*

**INPUT**: Set *ecosize*, create population of organisms *X*_*i*_, *i* = 1, 2, 3, …, *ecosize*, initialize *X*_*i*_, Set stopping criteria, Initialize SA parameters: Initial temperature *T*_*init*_, Final temperature *T*_*fin*_, Cooling rate *δ*.

**OUTPUT**: Optimal schedule

1:  Identify the best organism *X*_*best*_

2:   **While** stopping criterion is not met

3:    **For** *i* = 1 to *ecosize*

4:     **Mutualism Phase**

5:      Simulated annealing on *X*_*i*_ according to Eq (2) using Algorithm 3

6:      Simulated annealing on *X*_*j*_ according to Eq (3) using Algorithm 3

7:      Transform *X*_*i*_ and *X*_*j*_ using Eq (1)

8:     **Commensalism Phase**

9:      Simulated annealing on *X*_*i*_ according to Eq (7) using Algorithm 3

10:      Apply Eq (11) to reduce the temperature

11:      Transform *X*_*i*_ using Eq (1)

12:     **Parasitism Phase**

13:      Create *parasite*_*vector*

14:      Update *X*_*j*_ according to Eq (9)

15:      Transform *X*_*j*_ using Eq (1)

16:    Identify the best organism *X*_*best*_

17:   **End For**

18:  **End While**

## Problem Formulation

When tasks to be scheduled are received by Cloud Broker (CB), it queries Cloud Information Service (CIS) to identify the services required to execute the received tasks from the user and then schedules the tasks on the discovered services. For instance, if tasks {*T*_1_, *T*_2_, *T*_3_, …, *T*_*n*_} are submitted to CB in a given time interval. The processing elements (Virtual Machines) are heterogeneous having varied processing speeds and memory, indicating that a task executed on different Virtual Machines (VMs) will result in disparate execution cost. Suppose Virtual Machines {*M*_1_, *M*_2_, *M*_3_, …, *M*_*k*_} are available when the tasks are received by CB. The tasks are scheduled on the available VMs and execution of the tasks are done on the basis of First-Come First-Serve. Our aim is to schedule tasks on VMs in order to achieve high utilization with minimal makespan. As a result, Expected Time to Compute (ETC) of the tasks to be scheduled on each VM will be used by the proposed method to make schedule decisions. ETC values were determined using the ratio of millions instructions per second (MIPS) of a VM to length of the task [40, 41]. ETC values are usually represented in matrix form, where the number of tasks to be scheduled represents the rows of matrix and number of available VMs indicates the columns of the matrix. Each row of ETC matrix represents execution times of a given task for each VM, while each column represents execution times of each task on a given VM. Since our objective is to minimize the makespan by finding the best group of tasks to be executed on VMs. Let *C*_*ij*_, *i* = 1, 2, 3, …, *m*, *j* = 1, 2, 3, …, *n* be the execution time of executing *jth* task on *ith* VM where *m* the number of VMs is and *n* is the number of tasks. The fitness value of each organism is determined using Eq (13), which determines the strength of the level of adaptation of the organism to the ecosystem.

$$\begin{array}{c} objective\;function=max\{\sum_{i=1}^{m}\frac{f(M_{i})}{m}\} \end{array}$$ (13)

$$\begin{array}{c} f\left(M_{i}\right)=\frac{\mu }{makespan} \end{array}$$ (14)

$$\begin{array}{c} \mu =\sum_{i=1}^{m}\frac{\lambda_{i}}{m} \end{array}$$ (15)

$$\begin{array}{c} \lambda_{i}=\frac{T_{i}}{makespan} \end{array}$$ (16)

$$\begin{array}{c} makespan=max\{C_{jk}|j\in T,j=1,2,3,...,n;k\in M,k=1,2,3,...,m\} \end{array}$$ (17)

where *f*(*M*_*i*_) is the fitness value of virtual machine *i*; *μ* is the average utilization of virtual machines ready for execution of tasks and its essence is to support load balancing among VMs, *λ*_*i*_ defines the utilization of virtual machine *i*; *n* is the number of tasks and *m* is the number VMs.

### Performance metrics

The following metrics were used to evaluate the performance of the proposed method.

*Makespan*. Makespan is the time when the last task finished execution. Makespan is the most popular metric for measuring the performance of scheduling the methods. Lower makespan indicates efficient scheduling of task to virtual machines. Let *T*_*M*_*i*__(*ji*) be the execution time of processing set of tasks *j* on virtual machine *M*_*i*_, makespan is defined by Eq (18).

$$\begin{array}{c} makespan=max\{T_{M_{1}}\left(j1\right),T_{M_{2}}\left(j2\right),T_{M_{3}}\left(j3\right),...,T_{M_{n}}\left(jm\right)\} \end{array}$$ (18)

where *m* and *n* are the number of virtual machines and tasks respectively.

*Response Time* is the time from when the job arrives in a system to the time the first task is scheduled. Response time measures the time taken for scheduling algorithm to respond to a job. Response time is determined by Eq (19).

$$\begin{array}{c} ResponseTime=T_{firstrun}-T_{arrival} \end{array}$$ (19)

where *T*_*firstrun*_ is the finishing time of first task; *T*_*arrival*_ is the arrival of the first task.

*Degree of Imbalance*. Let *T*_*max*_, *T*_*min*_ and *T*_*avg*_ denotes the maximum, minimum, and average total execution times, respectively, among all VMs. Degree of imbalance (DI) defines the extent of load distribution among VMs according to their processing capacities, DI is determined by Eq (20).

$$\begin{array}{c} DI=\frac{T_{max}-T_{min}}{T_{avg}} \end{array}$$ (20)

### Simulation and Results

*Simulation Parameter Settings and Datasets*: The performance of the proposed method was evaluated using CloudSim [42]. CloudSim is a toolkit for modeling cloud computing scenarios. Two datacenters were created each containing two hosts respectively. Each host has 20 GB ram, 1 TB storage, 10 GB/s bandwidth and time-shared VM scheduling algorithm. One of the hosts is a dual-core machine while the other host is a quad-core machine each with X86 architecture, Linux operating system, Xen virtual machine monitor (VMM), and cumulative processing power of 1000000 MIPS. 25 VMs were created each with image size of 10 GB, 0.5 GB memory, 1 GB/s bandwidth and 1 processing element. The processing power of the VMs ranges from 100 to 5000 MIPS respectively. Time-shared cloudlet scheduler and Xen VMM were used for all the VMs. Six different data sets were used to evaluate the performance of the proposed method, four of which are generated using normal, left-skewed, right-skewed, and uniform distribution presented as S1, S2, S3 and S4 Datasets respectively. Uniform distribution depicts medium size tasks, and fewer small and large size tasks. Left-skewed represents fewer small size tasks and more large size tasks while right-skewed is the opposite. Uniform distribution depicts equal number of large, medium, and small size tasks. For each distribution, 100, 200, 300, 400, 500, 600, 700, 800, 900, 1000 instances were generated. The larger instances will enable us gain insight into the scalability of performance of the algorithms with large problem sizes. The parallel workloads used for evaluation are NASA Ames iPSC/860 [43] and HPC2N [44] presented as S5 and S6 Datasets respectively. NASA Ames iPSC/860 and HPC2N set log are some of the popular standard formatted workloads for evaluating the performance of distributed systems. NASA Ames iPSC/860 set log contains information of 14,794 tasks while HPC2N set log contains information of 527, 371 tasks. The simulation was run 30 times for each algorithm. The parameter settings of the algorithms are shown in Table 1.

**Table 1:** Parameter Settings of the Algorithms.

*Algorithm*	*Parameter*	*Value*
SOS	Number of Organisms	100
	Number of Iterations	1,000
SA	Initial temperature, *F*_*init*_	10
	Final temperature, *F*_*final*_	0.001
	Cooling rate, *δ*	0.9

**Fig 1:**
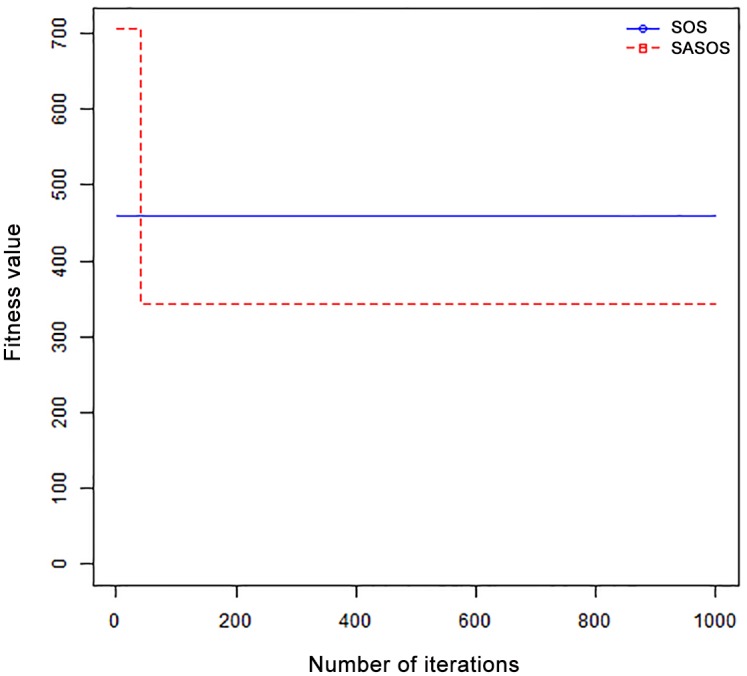
Convergence curve - Normal Dist - 100 Tasks.

**Fig 2:**
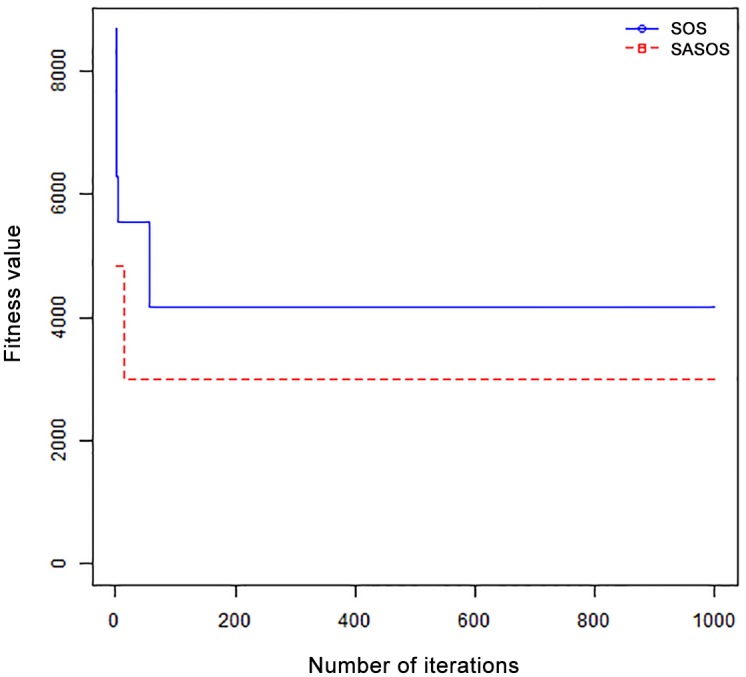
Convergence curve - Normal Dist - 500 Tasks.

**Fig 3:**
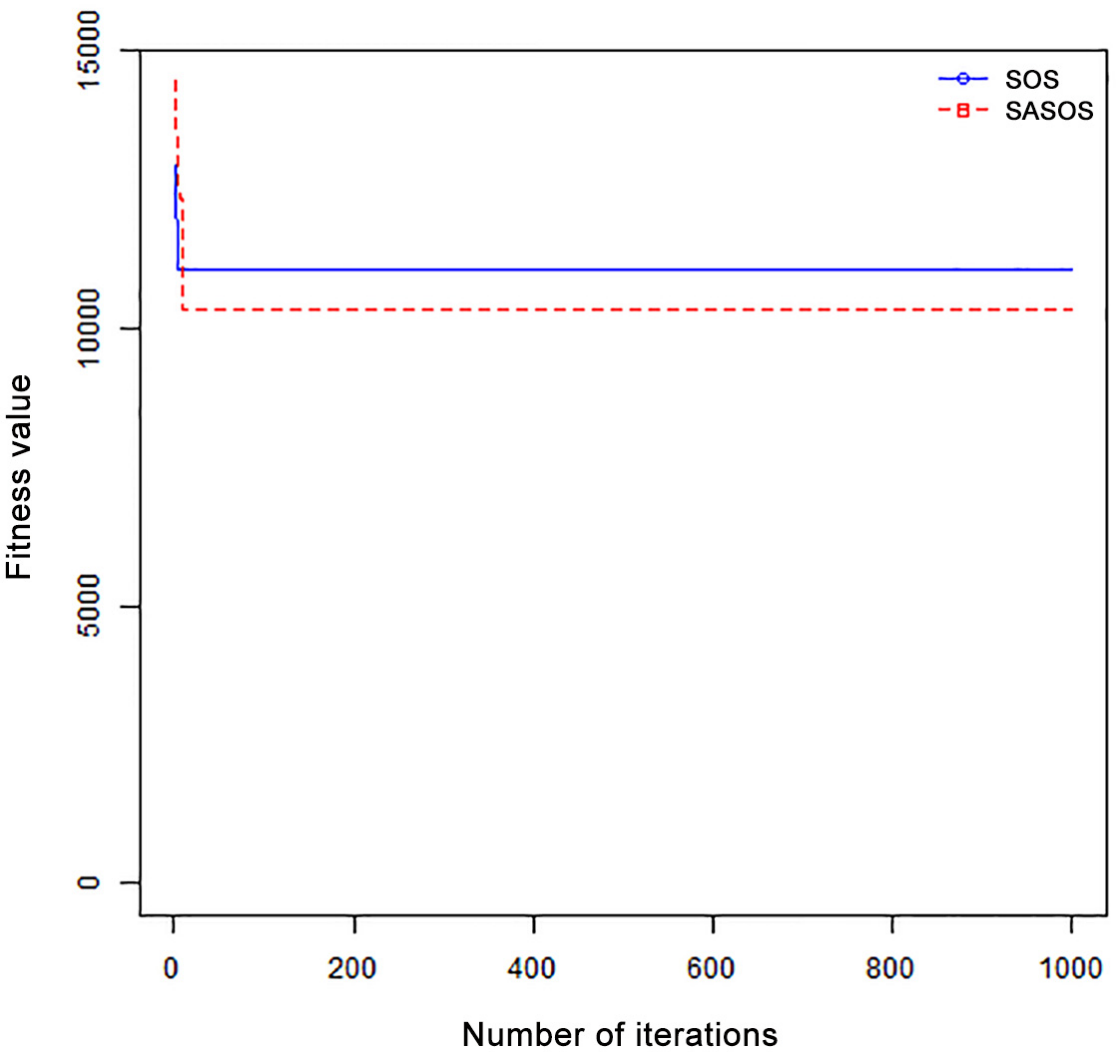
Convergence curve - Normal Dist - 1000 Tasks.

**Fig 4:**
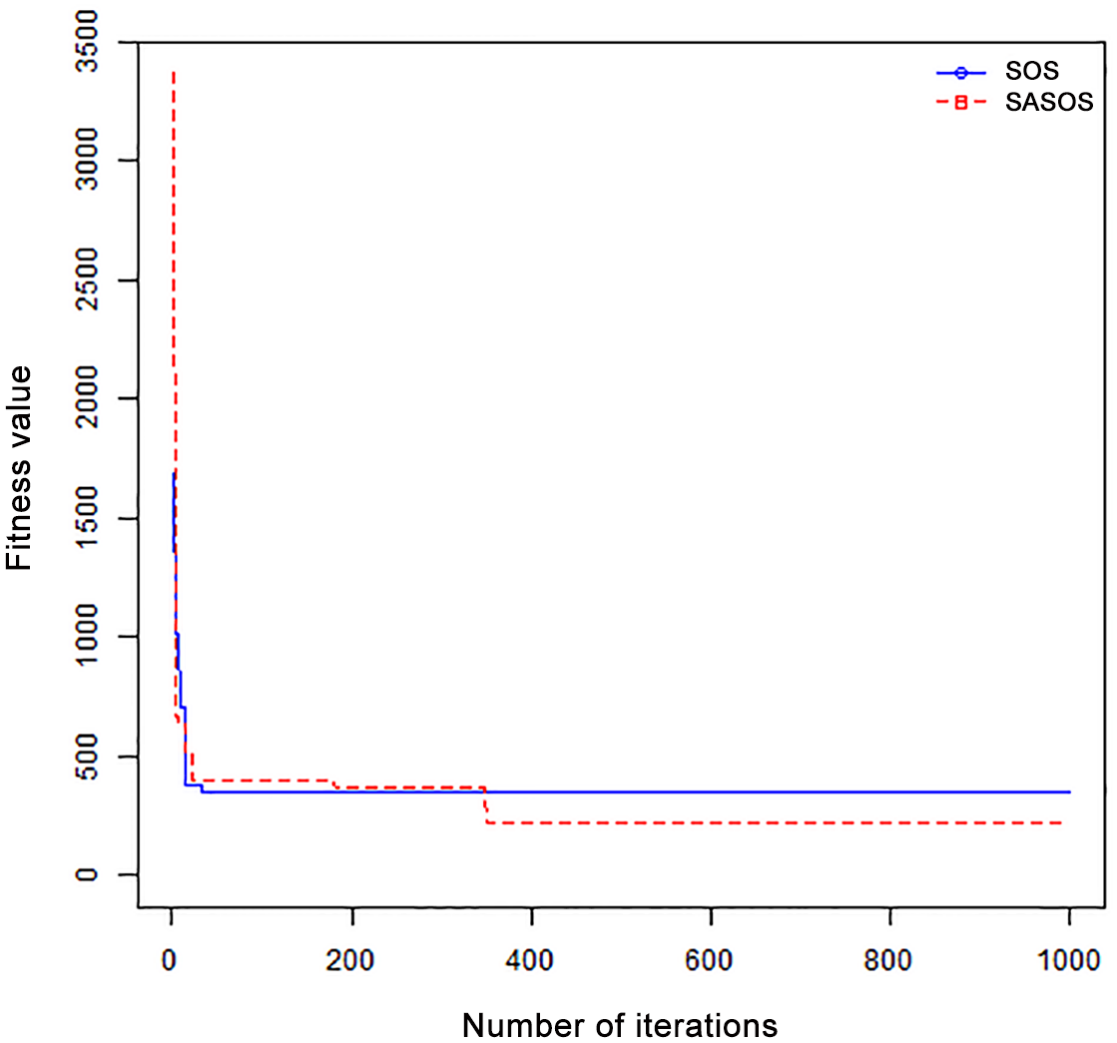
Convergence curve - Left Normal Dist - 100 Tasks.

**Fig 5:**
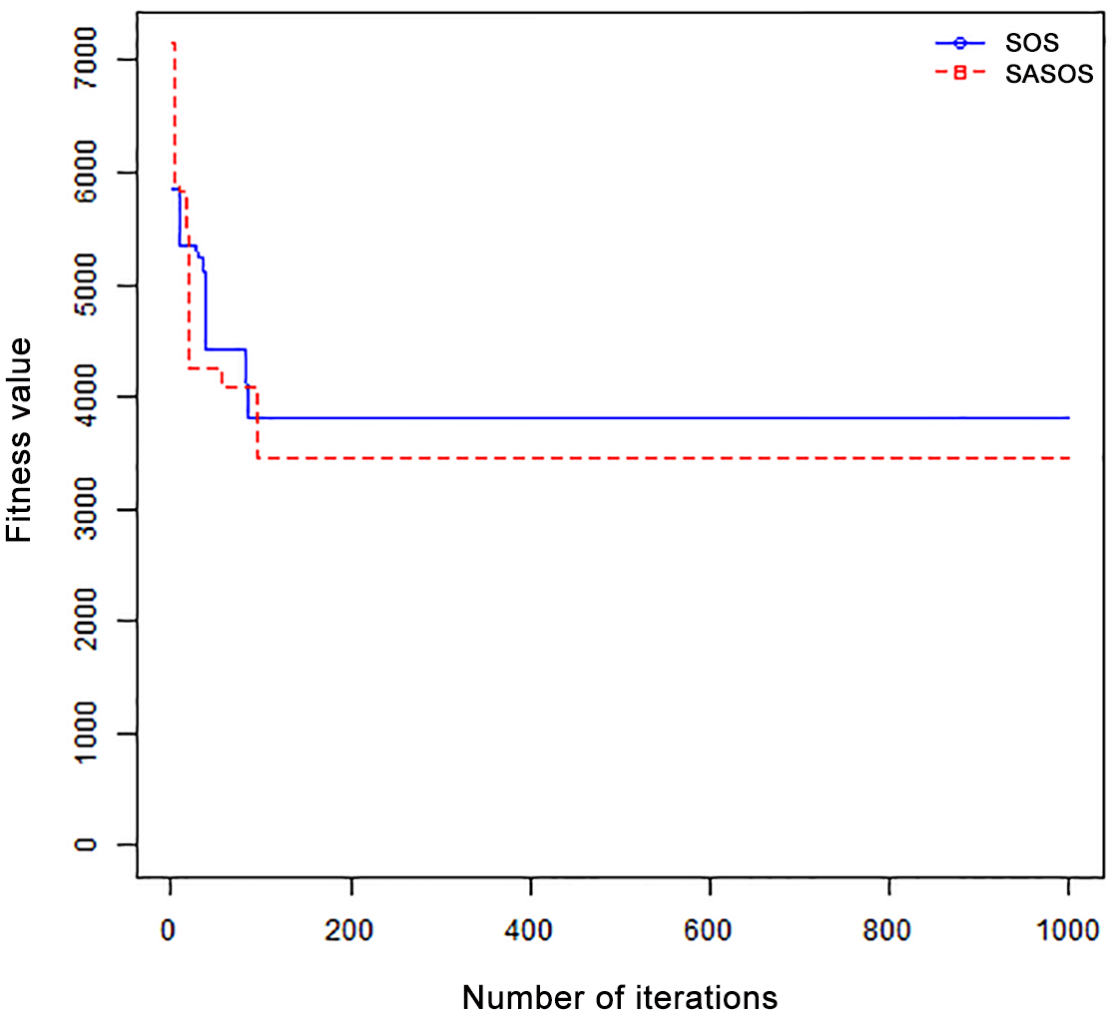
Convergence curve - Left Normal Dist - 500 Tasks.

**Fig 6:**
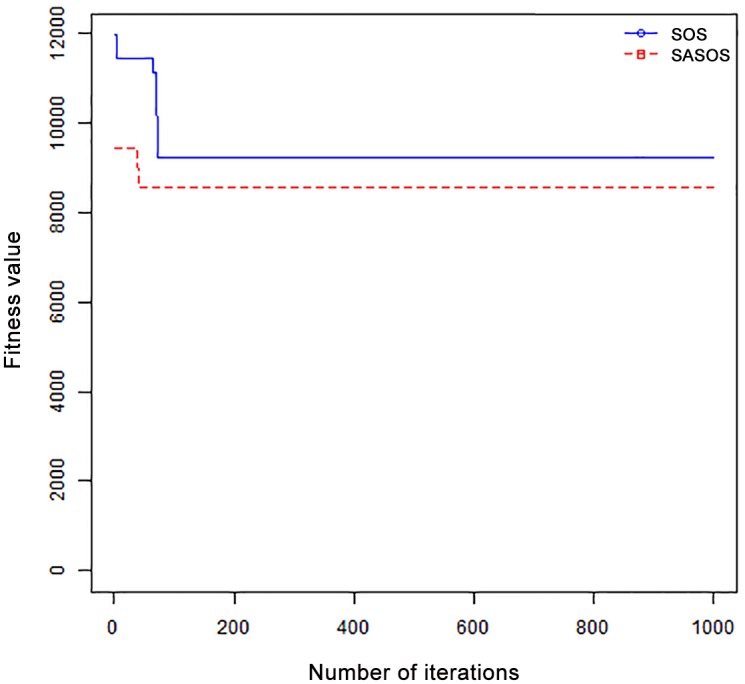
Convergence curve - Left Normal Dist - 1000 Tasks.

**Fig 7:**
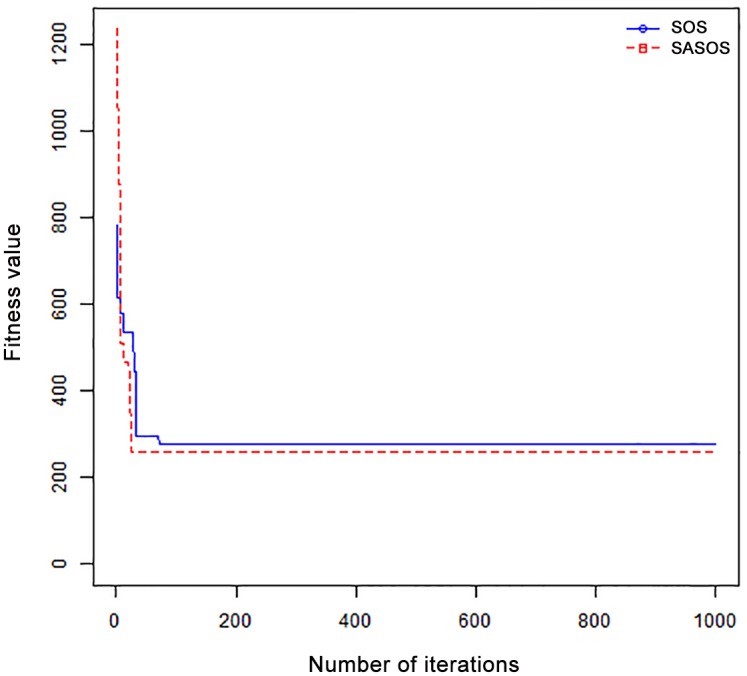
Convergence curve - Right Normal Dist - 100 Tasks.

**Fig 8:**
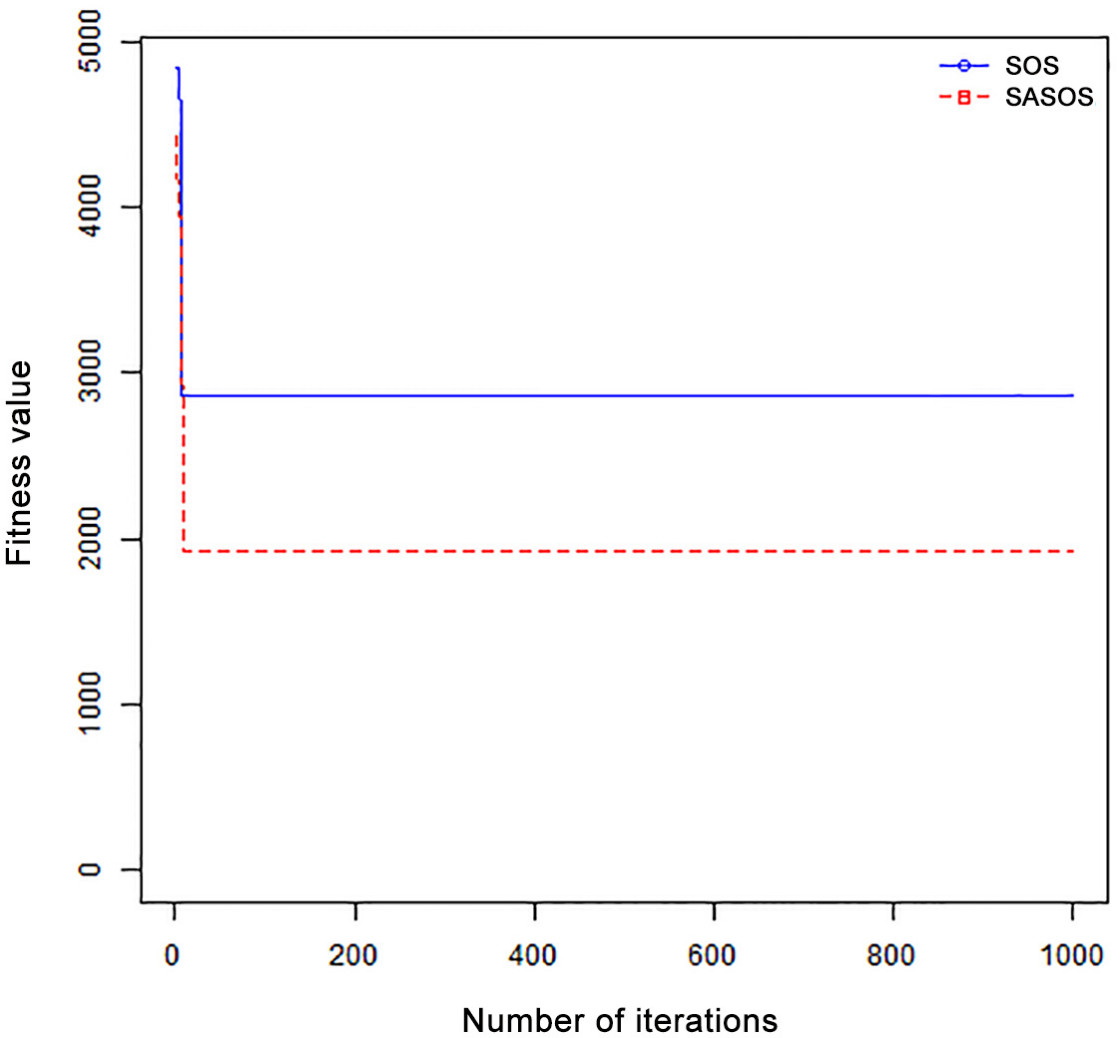
Convergence curve - Right Normal Dist - 500 Tasks.

**Fig 9:**
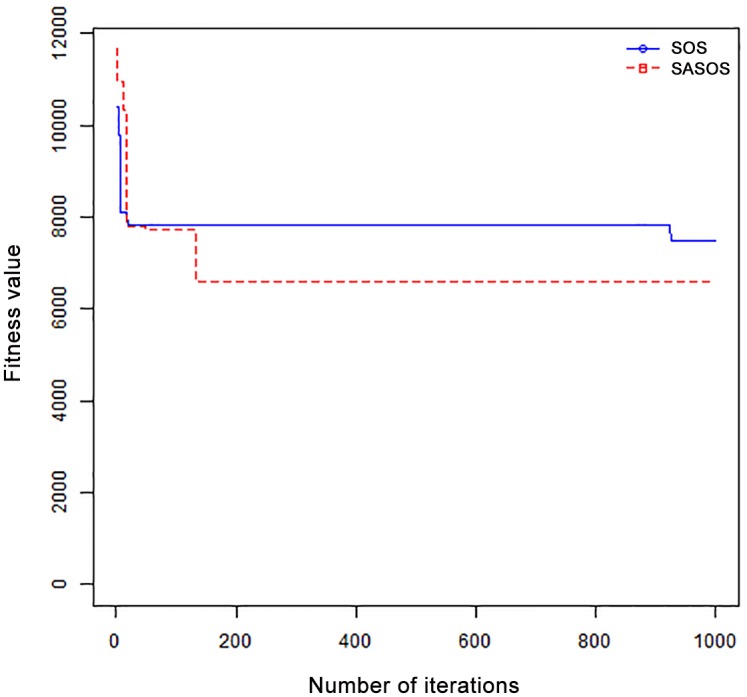
Convergence curve - Right Normal Dist - 1000 Tasks.

**Fig 10:**
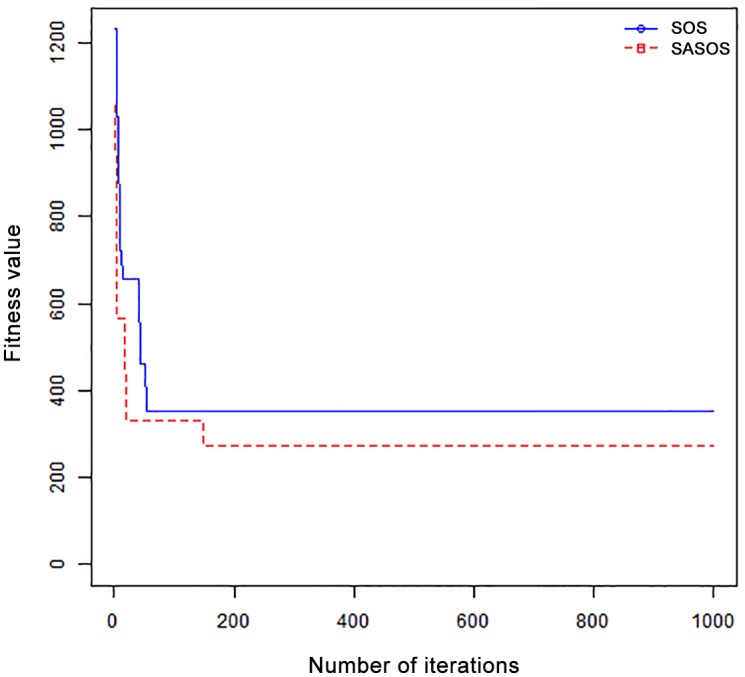
Convergence curve - Uniform Dist - 100 Tasks.

**Fig 11:**
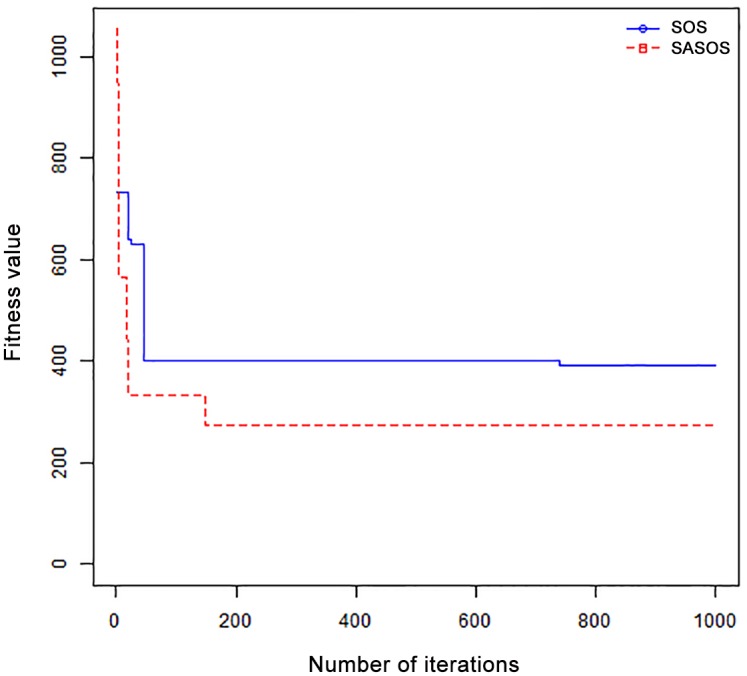
Convergence curve - Uniform Dist - 500 Tasks.

**Fig 12:**
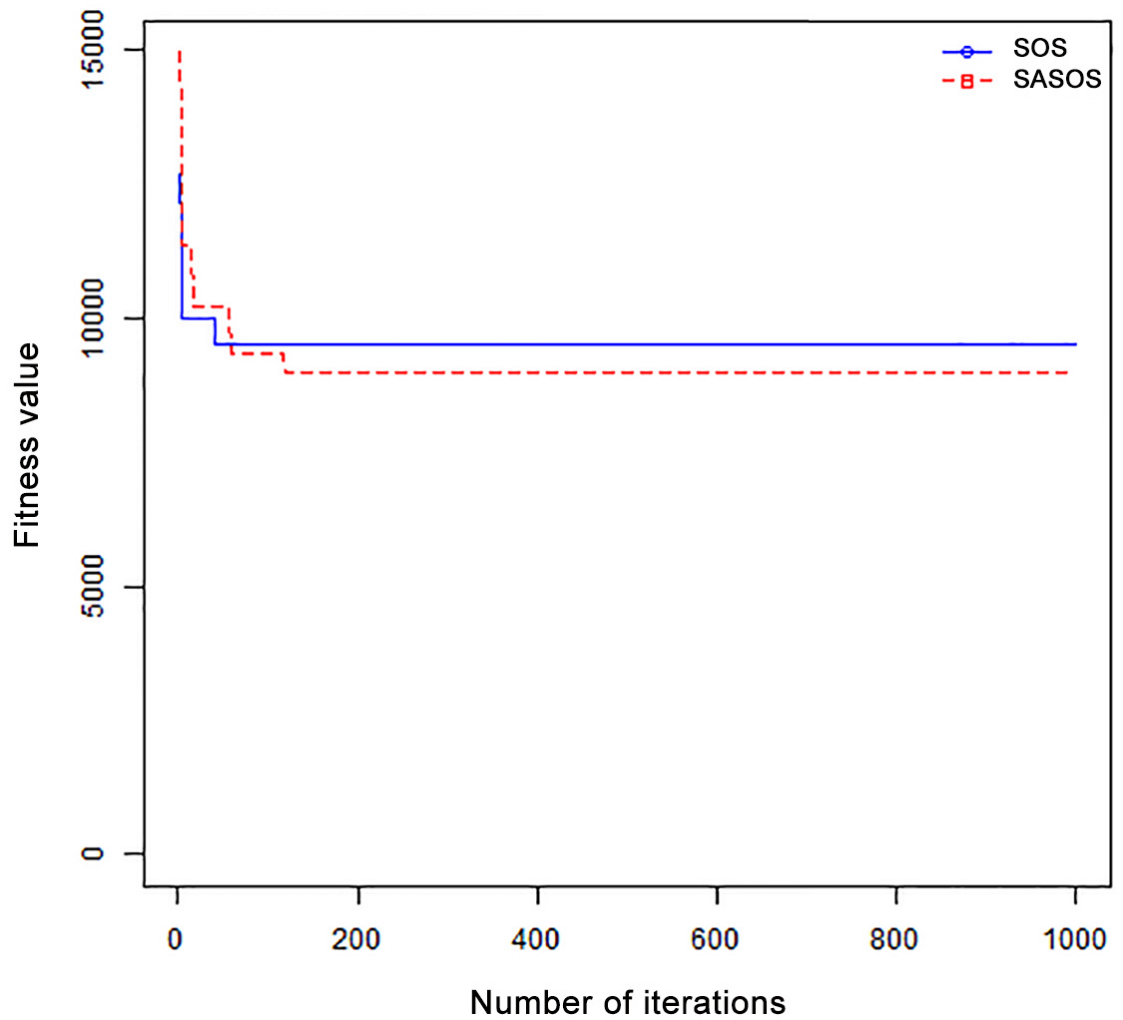
Convergence curve - Uniform Dist - 1000 Tasks.

**Fig 13:**
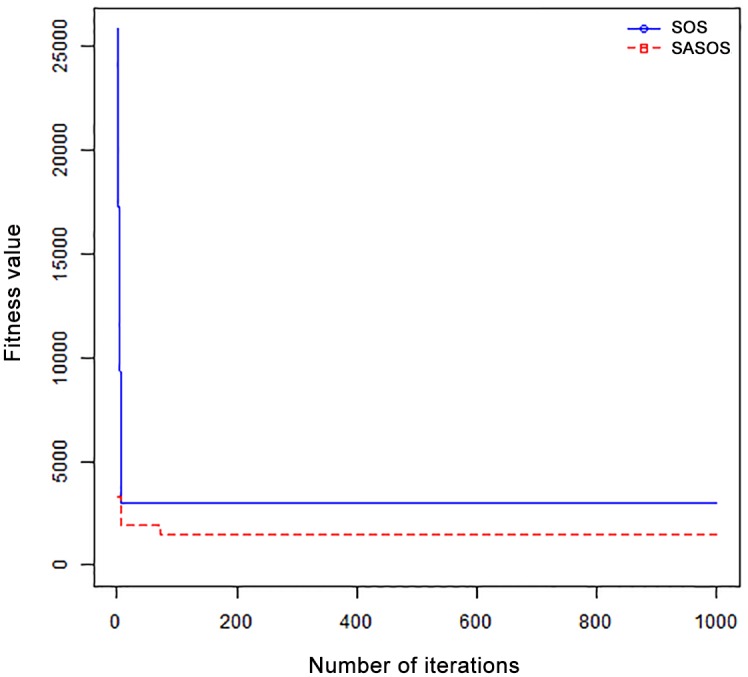
Convergence curve - HPC2N - 100 Tasks.

**Fig 14:**
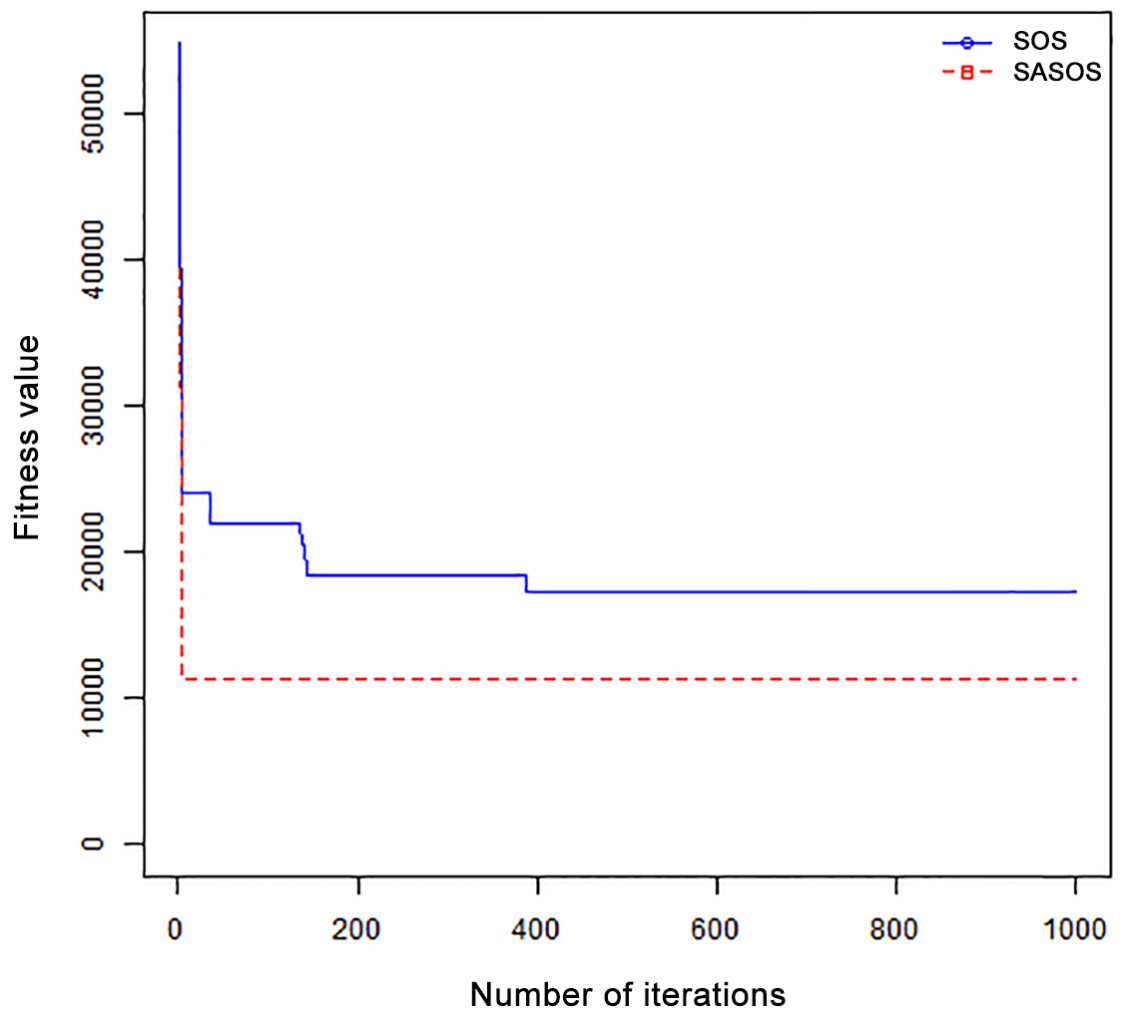
Convergence curve - HPC2N - 500 Tasks.

**Fig 15:**
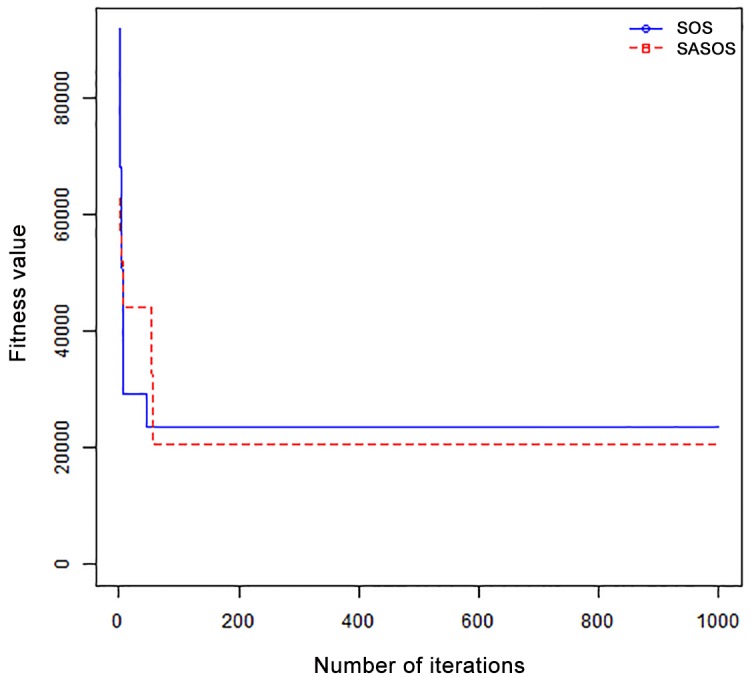
Convergence curve - HPC2N - 1000 Tasks.

**Fig 16:**
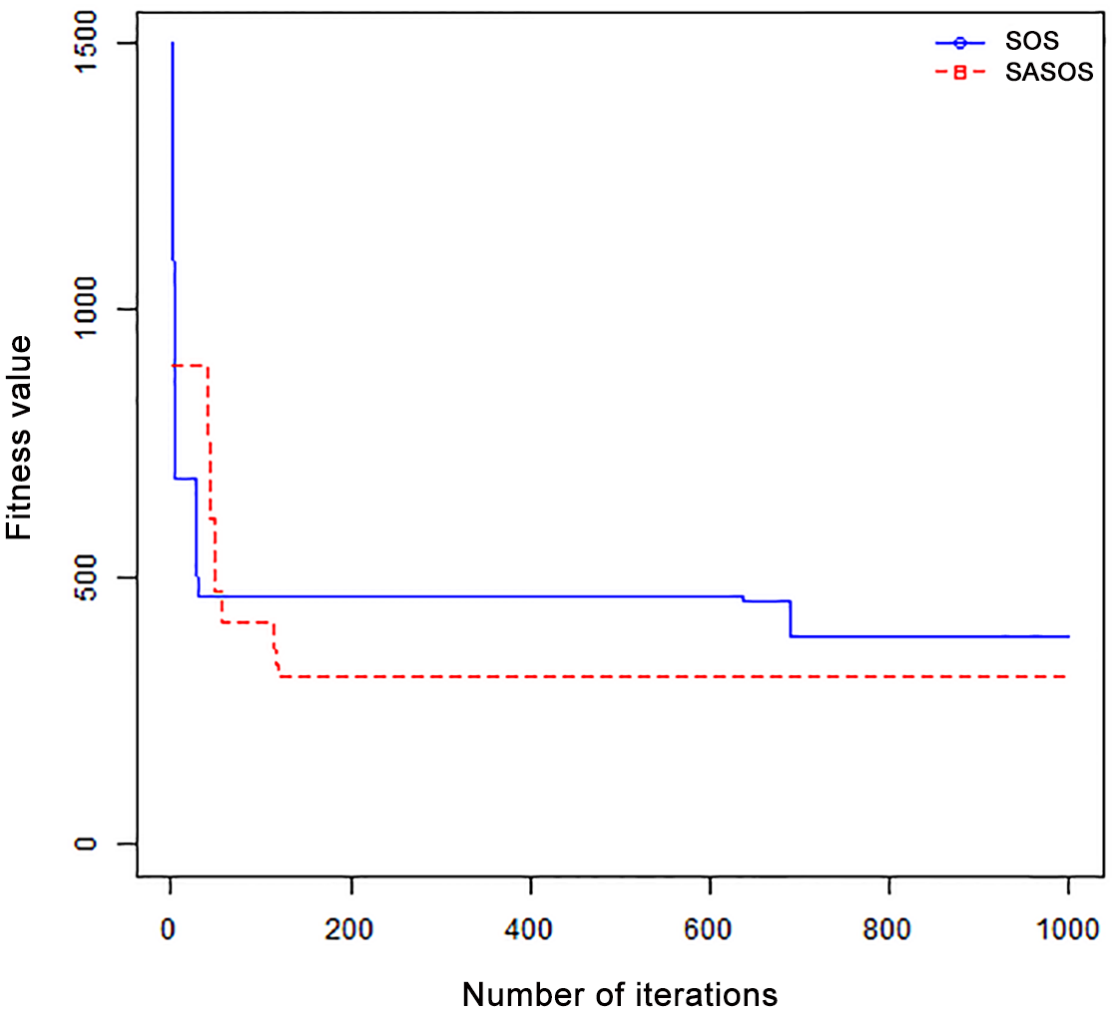
Convergence curve - NASA Ames iPSC/860 - 100 Tasks.

**Fig 17:**
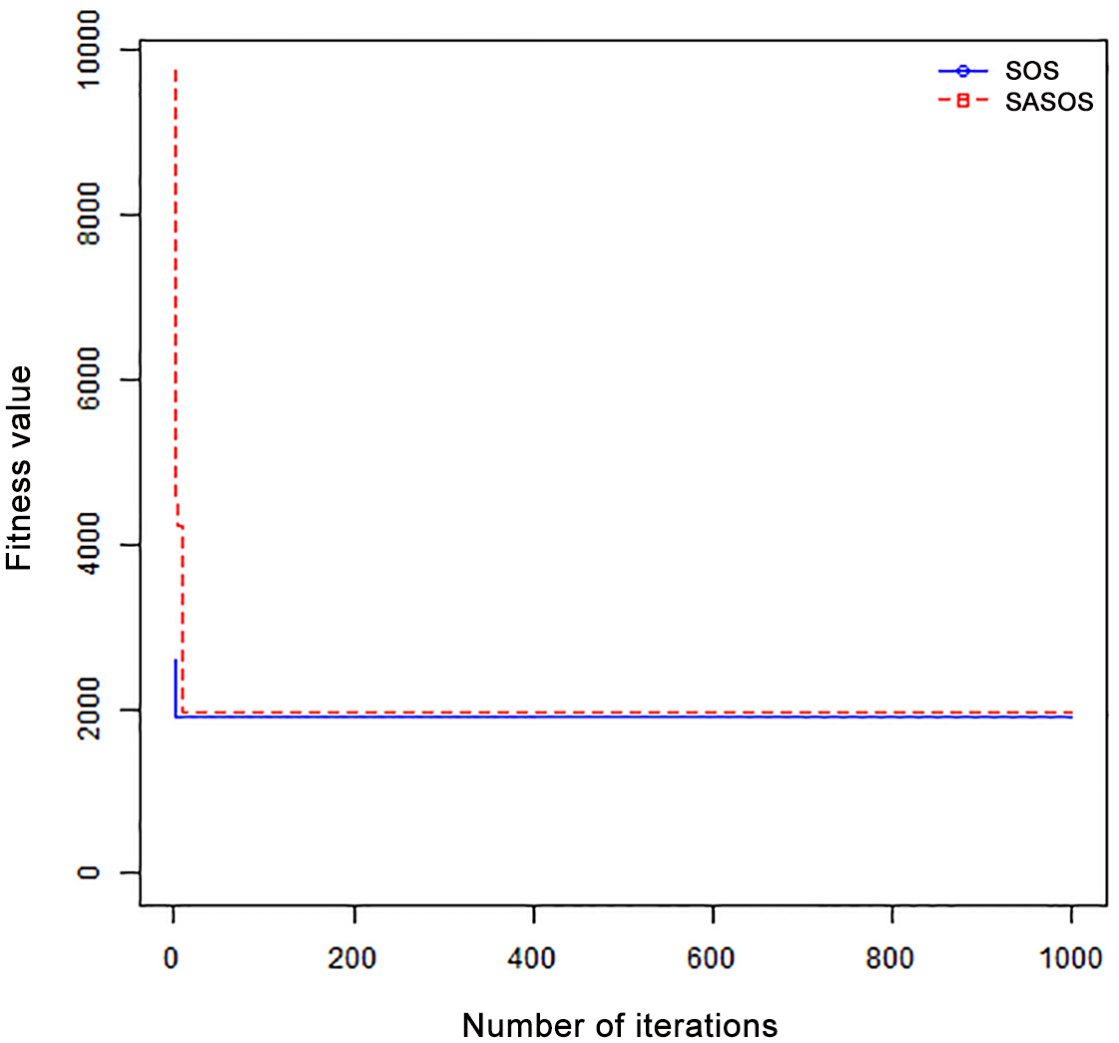
CConvergence curve - NASA Ames iPSC/860 - 500 Tasks.

**Fig 18:**
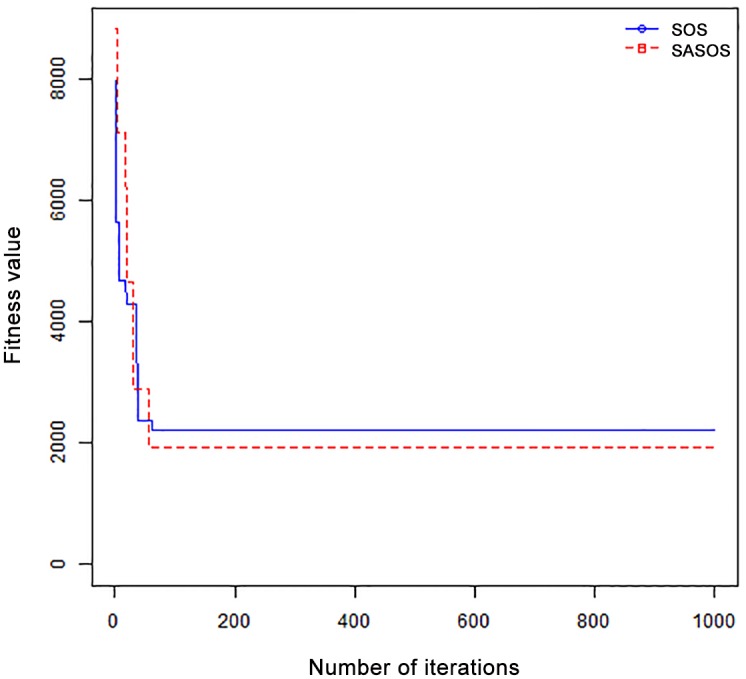
Convergence curve - NASA Ames iPSC/860 - 1000 Tasks.

*Results*: In order to exhibit the performance of SASOS against SOS, graphs of solution quality are plotted against number of iterations for the task sizes of 100, 500, and 1000 for the six data sets as shown in Figs 1–18. These graphs indicate variation in fitness values with respect to number of iterations. The plots depict the rate of convergence and speed at which the algorithms attain the optimal solution. To evaluate the algorithm that produces better solution, makespan, response time, and degree of imbalance are obtained using each algorithm. The values for these evaluation metrics are determined when applied to six different data sets as reported in Tables 2–18. The first column of each table indicates the number of tasks scheduled, the columns named “Average”, “Best”, and “Worst” reports the average, the best, and worst values obtained by each algorithm. The last column of each table record the improvement in average values obtained by SASOS with respect to SOS. For makespan, it can be observed that SASOS has better average makespan for all the six data sets as shown in Tables 2–7. For the response time, SASOS outperforms SOS in most cases of data sizes and data sets as shown in Tables 8–13. For degree of imbalance, SASOS produced better degree of imbalance among VMs as compared to SOS for all the task sizes and data sets as shown in Tables 14–19.

**Table 2:** Makespan - Normal Distributed Dataset.

*Number of Tasks*	SOS			SASOS			*Improvement(%)*
	*Average*	*Worst*	*Best*	*Average*	*Worst*	*Best*	
100	340.84	543.51	233.77	308.96	335.93	200.13	9.35
200	855.22	1154.44	513.55	833.60	936.26	618.87	2.53
300	1594.80	2195.89	934.64	1451.35	1533.16	1154.17	8.99
400	2485.28	3160.43	1767.26	2314.53	2408.34	1885.51	6.87
500	3392.88	4818.32	1907.79	3156.22	3397.93	1640.25	6.98
600	4566.58	5804.35	2704.00	4049.74	4175.89	3307.72	11.32
700	5402.63	7002.00	3585.27	4948.34	5262.50	3840.99	8.41
800	6762.20	8324.86	5193.88	6007.49	6326.44	4569.10	11.16
900	7775.22	9145.98	6132.36	7299.38	7614.49	5962.33	6.12
1000	9005.29	10666.64	6678.40	8116.80	8566.45	6754.41	9.87

305.0964857 273.791515 4172.53081 3007.850467 11049.81308 10339.47494

**Table 3:** Makespan - Left Normal Distributed Dataset.

*Number of Tasks*	SOS			SASOS			*Improvement(%)*
	*Average*	*Worst*	*Best*	*Average*	*Worst*	*Best*	
100	300.58	384.09	189.87	277.58	292.68	208.50	7.65
200	922.10	1337.91	605.07	862.92	939.22	552.30	6.42
300	1369.58	2031.05	940.35	1213.79	1316.38	943.04	11.38
400	2056.43	2861.88	1230.52	1966.89	2180.72	1351.94	4.35
500	3136.91	4196.15	2327.13	2905.63	3159.70	1974.60	7.37
600	4231.61	5452.52	2701.55	3819.96	4014.23	3179.25	9.73
700	4820.95	5804.18	3267.56	4398.35	4719.80	3603.35	8.77
800	6041.50	7622.91	3949.37	5454.02	5651.70	4495.87	9.72
900	6769.88	8227.34	4753.56	6526.76	6835.90	5663.86	3.59
1000	8105.26	9414.20	6242.02	7666.44	7940.73	6426.48	5.41

**Table 4:** Makespan - Right Normal Distributed Dataset.

*Number of Tasks*	SOS			SASOS			*Improvement(%)*
	*Average*	*Worst*	*Best*	*Average*	*Worst*	*Best*	
100	235.55	384.35	174.40	212.25	222.24	168.98	9.89
200	601.06	824.09	369.42	538.64	581.53	340.50	10.38
300	1190.98	1609.64	754.42	1071.18	1198.71	661.88	10.06
400	1735.90	2357.59	1152.43	1590.70	1727.16	1034.12	8.36
500	2487.28	3206.15	1652.03	2318.91	2482.31	1938.34	6.77
600	3283.42	4236.51	2347.23	2927.33	3132.88	2390.28	10.85
700	3871.93	4648.77	2334.26	3549.22	3757.03	2846.19	8.33
800	4871.12	5899.74	3000.00	4477.02	4718.91	3395.64	8.09
900	5516.24	6617.94	4034.83	4994.98	5167.92	4262.64	9.45
1000	6402.55	7889.11	5331.20	6037.42	6244.33	5207.99	5.70

**Table 5:** Makespan - Uniform Distributed Dataset.

*Number of Tasks*	SOS			SASOS			*Improvement(%)*
	*Average*	*Worst*	*Best*	*Average*	*Worst*	*Best*	
100	312.21	438.57	215.73	278.02	297.00	196.12	10.95
200	807.07	1115.35	507.72	734.91	817.32	522.65	8.94
300	1470.53	1904.61	859.45	1375.71	1484.38	1035.98	6.45
400	2334.28	3309.66	1654.35	1976.99	2168.68	1423.27	15.31
500	3263.91	4396.75	2244.82	2926.87	3099.04	2092.69	10.33
600	4201.65	5391.53	3094.80	3698.41	3847.80	2893.99	11.98
700	5023.15	6118.88	3561.55	4679.92	4956.90	3744.04	6.83
800	6157.11	8487.05	4369.69	5430.88	5800.05	3501.86	11.79
900	7106.45	8446.11	4874.59	6494.58	6825.56	5381.45	8.61
1000	8095.45	9880.74	6171.13	7641.47	7942.74	6680.93	5.61

**Table 6:** Makespan - HPC2N Dataset.

*Number of Tasks*	SOS			SASOS			*Improvement(%)*
	*Average*	*Worst*	*Best*	*Average*	*Worst*	*Best*	
100	2457.49	4206.35	1221.64	2070.16	2426.97	977.22	15.76
200	3920.99	6815.86	2520.10	3343.18	3671.80	2306.85	14.74
300	5705.85	10740.00	3850.24	5212.02	5625.56	3562.99	8.65
400	9600.07	18569.45	4629.71	6763.61	7696.66	4109.96	29.55
500	15661.89	29745.68	9872.91	11276.64	12011.90	6283.30	28.00
600	16635.51	36649.05	8877.67	14323.00	15345.44	8896.67	13.90
700	18989.38	33437.80	9515.80	14867.05	16777.16	8279.48	21.71
800	20871.05	30514.99	14058.66	18186.87	20535.14	10674.40	12.86
900	23508.46	41931.84	14683.98	20069.85	22081.20	11790.67	14.63
1000	26923.49	47122.40	15284.23	21631.11	23525.46	14043.55	19.66

**Table 7:** Makespan - NASA Ames iPSC/860 Dataset.

*Number of Tasks*	SOS			SASOS			*Improvement(%)*
	*Average*	*Worst*	*Best*	*Average*	*Worst*	*Best*	
100	400.09	848.48	291.07	368.51	385.74	296.79	7.89
200	471.03	891.53	292.80	387.81	421.73	292.29	17.67
300	1017.80	1699.46	459.23	854.96	906.35	523.83	16.00
400	1148.54	1845.14	561.17	997.83	1128.25	614.51	13.12
500	1379.16	2644.29	576.68	1209.09	1360.93	715.18	12.33
600	1659.51	2956.01	806.58	1372.54	1560.35	888.95	17.29
700	2015.81	3962.73	1124.88	1559.20	1838.23	966.60	22.65
800	2173.10	3547.90	804.46	1645.06	1824.72	1049.74	24.30
900	2672.28	5665.10	1399.80	2160.63	2456.35	1224.57	19.15
1000	2874.95	6116.32	1349.54	2332.00	2540.76	1266.85	18.89

**Table 8:** Response Time - Normal Distributed Dataset.

*Number of Tasks*	SOS			SASOS			*Improvement(%)*
	*Average*	*Worst*	*Best*	*Average*	*Worst*	*Best*	
100	3.36	4.42	2.75	3.23	3.76	2.46	4.06
200	4.02	4.77	2.71	4.13	5.43	3.09	-2.9
300	4.68	6.04	3.03	4.52	5.67	3.56	3.33
400	5.27	6.33	3.88	5.19	6.14	4.2	1.59
500	5.6	7.08	3.54	5.59	6.92	3.04	0.1
600	6.06	7.21	4.02	5.83	7	4.73	3.78
700	6.18	7.25	4.44	5.99	7.29	4.81	3.06
800	6.63	7.4	5.54	6.29	7.44	4.94	5.07
900	6.69	7.46	5.59	6.59	7.46	5.55	1.47
1000	6.86	7.57	5.31	6.58	7.64	5.4	4.02

**Table 9:** Response Time - Left Normal Distributed Dataset.

*Number of Tasks*	SOS			SASOS			*Improvement(%)*
	*Average*	*Worst*	*Best*	*Average*	*Worst*	*Best*	
100	3.38	4.36	2.72	3.24	3.74	2.78	4.03
200	4.23	5.39	3.06	4.19	5.74	2.84	1
300	4.62	5.84	3.25	4.46	5.84	3.37	3.32
400	5.02	6.29	3.57	5.11	6.04	3.59	-1.83
500	5.63	6.56	4.3	5.55	6.7	3.82	1.41
600	5.97	7.11	4.28	5.81	7.3	4.7	2.77
700	6.16	6.97	4.67	5.88	6.86	4.71	4.6
800	6.46	7.62	4.59	6.27	7.32	5.26	2.85
900	6.7	7.39	5.16	6.62	7.59	5.66	1.11
1000	6.82	7.53	5.54	6.69	7.57	5.66	1.91

**Table 10:** Response Time - Right Normal Distributed Dataset.

*Number of Tasks*	SOS			SASOS			*Improvement(%)*
	*Average*	*Worst*	*Best*	*Average*	*Worst*	*Best*	
100	3.18	4.24	2.44	3.03	4.04	2.4	4.59
200	3.87	4.81	2.55	3.85	5.16	2.69	0.68
300	4.64	5.78	3.25	4.58	5.61	3.1	1.13
400	5.11	6.21	3.65	5.02	6.41	3.39	1.8
500	5.6	6.68	4.09	5.53	6.7	4.54	1.29
600	5.91	6.9	4.42	5.72	6.81	4.63	3.29
700	6.04	7.31	4.17	6.07	7.49	4.78	-0.51
800	6.52	7.37	4.4	6.32	7.5	4.84	3
900	6.68	7.37	5.2	6.4	7.46	5.36	4.23
1000	6.78	7.76	5.91	6.67	7.52	5.71	1.55

**Table 11:** Response Time - Uniform Distributed Dataset.

*Number of Tasks*	SOS			SASOS			*Improvement(%)*
	*Average*	*Worst*	*Best*	*Average*	*Worst*	*Best*	
100	3.31	4.11	2.47	3.19	3.92	2.62	3.61
200	4.03	5.27	2.78	4.04	5.15	2.9	-0.37
300	4.61	5.57	2.84	4.66	5.66	3.46	-1.03
400	5.3	6.46	3.99	4.86	6.14	3.49	8.3
500	5.65	6.6	4.26	5.42	6.67	3.9	4.13
600	6.12	7.05	4.86	5.81	6.94	4.46	5.06
700	6.24	7.03	4.84	6.06	7.49	4.79	2.97
800	6.45	7.48	4.91	6.15	7.1	4.24	4.6
900	6.59	7.59	4.82	6.42	7.51	5.33	2.59
1000	6.77	7.71	5.5	6.68	7.5	5.8	1.38

**Table 12:** Response Time - HPC2N Dataset.

*Number of Tasks*	SOS			SASOS			*Improvement(%)*
	*Average*	*Worst*	*Best*	*Average*	*Worst*	*Best*	
100	3.04	3.63	2.41	3.02	3.55	2.56	0.77
200	2.89	3.53	2.16	2.85	3.67	2.29	1.48
300	2.93	3.67	2.26	2.96	3.76	2.31	-1.02
400	3.21	4.15	2.37	2.95	4.48	1.98	8.26
500	3.36	4.19	2.57	3.22	4.13	2.27	3.97
600	3.54	4.37	2.42	3.34	4.69	2.58	5.5
700	3.62	4.84	2.54	3.47	4.71	2.14	4.19
800	3.71	4.7	2.4	3.66	5.13	2.45	1.41
900	3.95	4.97	2.65	3.87	4.83	2.48	1.95
1000	4.05	4.94	3.04	3.91	4.83	3.02	3.54

**Table 13:** Response Time - NASA Ames iPSC/860 Dataset.

*Number of Tasks*	SOS			SASOS			*Improvement(%)*
	*Average*	*Worst*	*Best*	*Average*	*Worst*	*Best*	
100	4.06	5.37	2.92	4.09	6.07	3.18	-0.6
200	3.55	5.01	1.98	3.35	4.53	2.11	5.72
300	3.01	3.81	2.15	3.03	3.81	2.35	-0.82
400	2.92	3.79	2.18	2.96	3.77	2.27	-1.55
500	3.07	3.93	2.2	3.02	4.63	2.36	1.78
600	3.22	3.77	2.12	3.03	3.99	2.3	5.92
700	3.18	4.14	2.52	3.03	3.68	2.29	4.65
800	3.33	4.39	2.3	3.23	4.1	2.76	2.85
900	3.36	4.25	2.43	3.27	4.53	2.53	2.61
1000	3.55	4.89	2.38	3.36	4.33	2.29	5.32

**Table 14:** Degree of Imbalance - Normal Distributed Dataset.

*Number of Tasks*	SOS			SASOS			*Improvement(%)*
	*Average*	*Worst*	*Best*	*Average*	*Worst*	*Best*	
100	12.77	26.04	12.59	9.53	29.18	12.89	25.43
200	28.63	47.48	29.4	14.57	50.67	22.59	49.11
300	45.6	68.71	49.79	34.37	77.64	49.79	24.62
400	64.19	106.52	70.7	47.67	113.28	68.34	25.74
500	80.84	138.79	88.59	53.19	124.66	76.54	34.21
600	96.35	149.99	101.25	68.74	145.43	87.87	28.65
700	129.81	203.91	143.26	88.16	186.14	131.16	32.08
800	147.12	216.53	158.24	98.06	196.59	137.63	33.35
900	161.53	232.68	170.1	125.46	248.74	180.34	22.33
1000	153.19	251.79	149.53	119.7	238.23	170.61	21.86

**Table 15:** Degree of Imbalance - Left Normal Distributed Dataset.

*Number of Tasks*	SOS			SASOS			*Improvement(%)*
	*Average*	*Worst*	*Best*	*Average*	*Worst*	*Best*	
100	10.7	23.22	9.42	7.68	21.7	9.31	28.25
200	25.92	51.61	24.6	16.93	49.51	25.99	34.67
300	35.65	71.01	35.79	24.96	68.93	43.54	29.98
400	52.27	98.96	55.29	34.77	89.51	51.63	33.49
500	79.92	123.28	85.92	57.27	124.41	86.06	28.35
600	107.35	158.83	112.95	65.36	157.88	96.54	39.11
700	107.81	169.17	105.38	86.1	162.52	124.69	20.13
800	145.88	214.83	152.11	98.21	197.23	144.29	32.67
900	150.59	244.38	161.7	95.53	217.15	149.38	36.56
1000	163.55	233.29	162.37	106.25	250.83	164.11	35.03

**Table 16:** Degree of Imbalance - Right Normal Distributed Dataset.

*Number of Tasks*	SOS			SASOS			*Improvement(%)*
	*Average*	*Worst*	*Best*	*Average*	*Worst*	*Best*	
100	8.47	16.56	8.26	6.5	17.93	8.86	23.34
200	18.1	31.88	17.71	11.98	28.66	16.67	33.83
300	26.73	45.38	25.82	19.87	48.63	30.86	25.69
400	40.67	63.54	41.1	31.97	67.24	42.66	21.39
500	47.51	75.74	50.87	34.19	85.33	54.36	28.04
600	59.57	93.72	60.89	48.76	100.72	67.57	18.13
700	74.53	111.89	79.11	55.93	102.1	80.99	24.95
800	84.58	133.52	88.72	64.47	130.93	100.34	23.78
900	99.25	158	107.79	65.14	144.16	99.28	34.37
1000	119.15	168.09	129.98	76.8	159.12	124.48	35.55

**Table 17:** Degree of Imbalance - Uniform Distributed Dataset.

*Number of Tasks*	SOS			SASOS			*Improvement(%)*
	*Average*	*Worst*	*Best*	*Average*	*Worst*	*Best*	
100	10.94	17.58	10.71	8.47	20.08	11.04	22.61
200	22	43.28	22.71	14.81	41.38	21.59	32.67
300	38.45	66.91	41.26	28.7	62.57	40.68	25.37
400	50.24	86.57	53.19	31.19	86.84	52.58	37.91
500	67.13	103.88	73.45	54.62	112.19	77.6	18.64
600	76.1	120.36	80.93	53.28	121.42	80.47	29.99
700	103.03	148.15	108.92	66.26	150.92	104.74	35.69
800	111.35	167.11	121.6	70.69	174.31	101.93	36.51
900	133.56	191.2	139.55	98.65	181.62	143.7	26.14
1000	139.3	196.2	149.77	120.5	218.16	163.06	13.49

**Table 18:** Degree of Imbalance - HPC2N Dataset.

*Number of Tasks*	SOS			SASOS			*Improvement(%)*
	*Average*	*Worst*	*Best*	*Average*	*Worst*	*Best*	
100	0.6218	2.1045	0.5437	0.3726	2.2741	0.6853	40.0763
200	0.1006	0.1208	0.1100	0.0818	0.1207	0.1100	18.6708
300	0.1105	0.1209	0.1100	0.1031	0.1208	0.1100	6.6970
400	0.1072	0.1209	0.1100	0.1065	0.1207	0.1100	0.6576
500	0.1098	0.1208	0.1100	0.1069	0.1194	0.1100	2.6107
600	0.1122	0.2200	0.1100	0.1074	0.2116	0.1100	4.2359
700	0.1144	0.2149	0.1100	0.1068	0.2200	0.1100	6.6075
800	0.1320	0.2208	0.1100	0.1080	0.2200	0.1100	18.1776
900	0.1635	0.2260	0.2067	0.1213	0.2233	0.2105	25.8238
1000	0.1899	0.2225	0.2139	0.1737	0.2268	0.2142	8.5132

**Table 19:** Degree of Imbalance - NASA Ames iPSC/860 Dataset.

*Number of Tasks*	SOS			SASOS			*Improvement(%)*
	*Average*	*Worst*	*Best*	*Average*	*Worst*	*Best*	
100	0.0378	0.1121	0.0054	0.0022	0.1127	0.0040	94.2187
200	0.0742	0.1123	0.1100	0.0088	0.1154	0.1058	88.1108
300	0.1066	0.2221	0.1100	0.0760	0.2242	0.1100	28.7243
400	0.0020	0.0038	0.0020	0.0015	0.0044	0.0019	24.1417
500	0.0021	0.0070	0.0020	0.0014	0.0039	0.0018	32.1536
600	0.0020	0.0050	0.0019	0.0016	0.0043	0.0020	21.3347
700	0.0020	0.0060	0.0019	0.0014	0.0034	0.0019	27.3566
800	0.0020	0.0033	0.0019	0.0015	0.0033	0.0019	25.4827
900	0.0020	0.0034	0.0019	0.0015	0.0044	0.0019	24.0295
1000	0.0020	0.0033	0.0019	0.0017	0.0069	0.0020	14.4083

From the convergence curve of normal distributed data sets shown in Figs 1–3, SOS converges faster to a stable state than SASOS for 100 tasks. SOS converges slower than SASOS for 500 tasks, while for 1000 tasks, both SOS and SASOS converges at the same rate. The convergence curves of left normal distributed data sets, Figs 4 and 5 indicate that SOS converges faster than SASOS but SOS is only able to locate local optima. For 1000 tasks SASOS converges faster than SOS as shown in Fig 6. SASOS converge faster than SOS for 100 and 1000 task sizes for right normal distributed data set as shown in Figs 7 and 9. Both SOS and SASOS converges at the same rate as shown in Fig 8. For uniform distributed data set, SOS algorithm converges faster than SASOS for 100, 500, and 1000 task sizes as shown in Figs 10–12. For HPC2N, SOS converges faster than SASOS for 100 and 1000 task sizes as depicted in Figs 13 and 15, while for 500 tasks SASOS converges faster than SOS as shown in Fig 11. For NASA Ames iPSC/860, SASOS converges faster than SOS for 100 tasks as shown in Fig 16, whereas SOS and SASOS converges at virtually the same point.

Furthermore, the search direction of SASOS algorithm converges to stable in less than 100 iterations in all cases except in one scenario as depicted in Fig 4 where the algorithm converges in about 300 iterations. SOS algorithm converges in most situations except few cases as shown in Figs 9, 11, 14 and 16. Fig 9 indicates that SOS converges after 900 iterations while Fig 11 showed that SOS converges at about 750 iterations. The search direction of SOS converges at about 400 iterations in the case of Fig 14, SOS converges at about 700 iterations in Fig 16 scenario. Moreover, the quality solutions obtained by SASOS algorithms are better than those of SOS, and SASOS search direction of SASOS tends to converge to a stable point in a lesser number of iterations. This performance could be attributed to the local search ability of SA employed into SOS. In addition, SOS has shown that it can improve its quality of solutions even at latter stage of search procedure as demonstrated in Figs 9, 11, 14 and 16. This could be attributed to parasitism phase of the algorithm which perturb the solution space eliminating inactive solution and introducing the active ones.

## Conclusion

This study presents a hybrid Symbiotic Organisms Search (SOS) algorithm named SASOS to obtain optimal schedule of tasks in cloud computing environment. The proposed algorithm employs local search search ability of Simulated Annealing (SA) in order to improve the speed of convergence and quality of solution obtained by SOS algorithm in terms of makespan, response time, and degree of imbalance. The simulation results show that SASOS outperforms SOS in terms of convergence rate and quality of solution. The proposed method can be used to solve other optimization issues in the cloud computing system and other discrete optimization problems in different domains. In the future, we intend focus on hybridizing SOS with other effective local search and metaheuristic techniques.

## Supporting Information

S1 Dataset Normal. (ZIP)

S2 Dataset Left-skewed. (ZIP)

S3 Dataset Right-skewed. (ZIP)

S4 Dataset Uniform. (ZIP)

S5 Dataset NASA Ames iPSC-860. (ZIP)

S6 Dataset HPC2N. (ZIP)
