# Correction: Hybrid Symbiotic Organisms Search Optimization Algorithm for Scheduling of Tasks on Cloud Computing Environment

**DOI:** 10.1371/journal.pone.0162054

**Published:** 2016-08-24

**Authors:** Mohammed Abdullahi, Md Asri Ngadi

The affiliation for the second author is incorrect. Md Asri Ngadi is not affiliated with #2 but with #1 Department of Computer Science, Universiti Teknologi Malaysia, 81310 Johor Bahru, Malaysia.
